# Supplementary material for: Optimal minimal residual disease threshold in pediatric acute myeloid leukemia: A retrospective cohort study based on the TARGET database
Source: PLoS Med. 2026 May 8;23(5):e1005088. doi: 10.1371/journal.pmed.1005088 (PMC13155632; doi:10.1371/journal.pmed.1005088)
Supplement: S1 Code — (ZIP) [file pmed.1005088.s002.zip › S2 code/PROJ8_22_tbl1/PROJ8_22_tbl1_seg2_b.pdf]

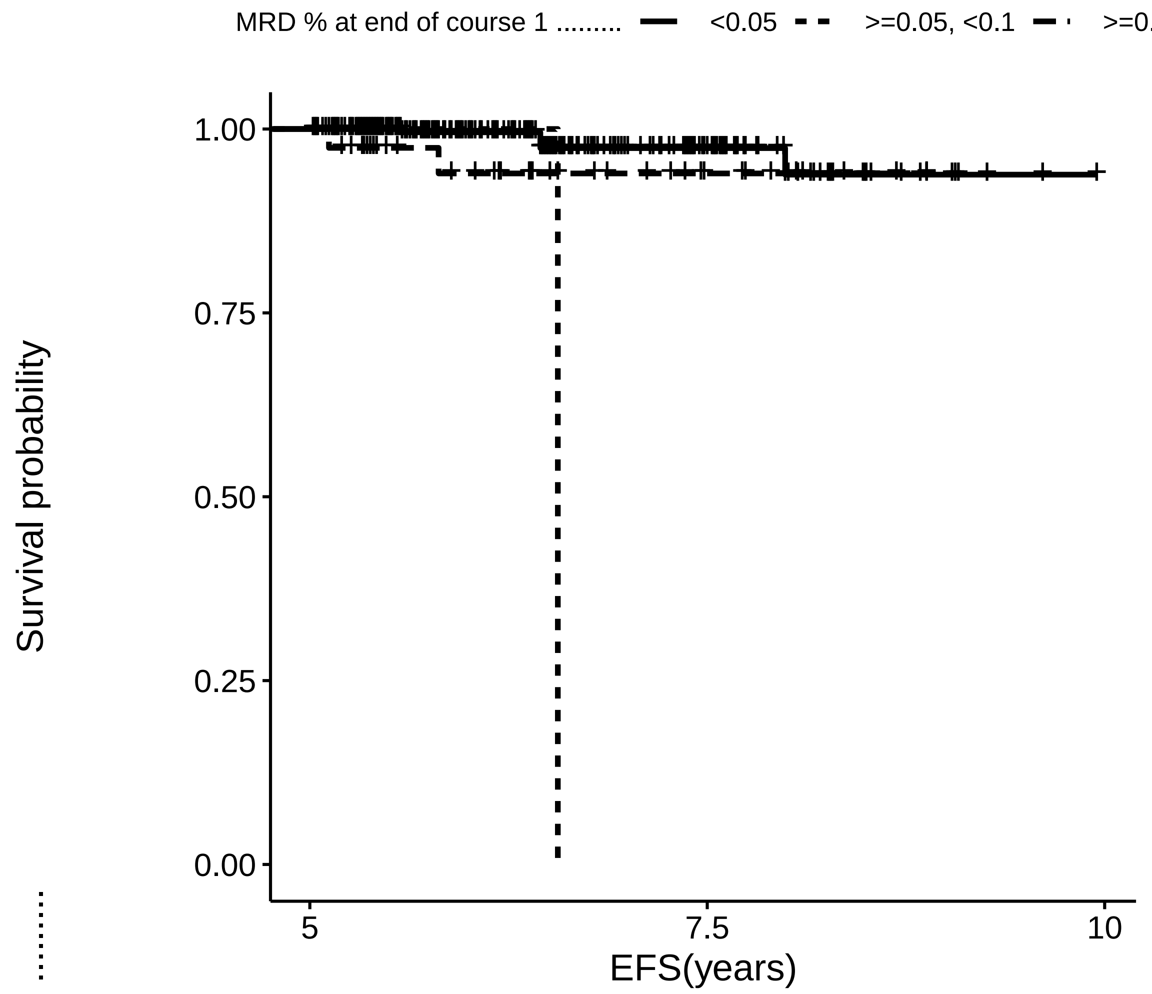

Number at risk

MRD % at end of course 1	5	6	7	8	9	10
<0.05	297	180	85	25	7	0
>=0.05, <0.1	1	1	0	0	0	0
>=0.1	39	26	15	5	0	0

EFS(years)
